# Relationship between Entropy and Dimension of Financial Correlation-Based Network

**DOI:** 10.3390/e20030177

**Published:** 2018-03-07

**Authors:** Chun-xiao Nie, Fu-tie Song

**Affiliations:** Department of Finance, School of Business, East China University of Science and Technology, Shanghai 200237, China

**Keywords:** fractal dimension, Rényi index, minimum spanning tree (MST), planar maximally filtered graph (PMFG), finance

## Abstract

We analyze the dimension of a financial correlation-based network and apply our analysis to characterize the complexity of the network. First, we generalize the volume-based dimension and find that it is well defined by the correlation-based network. Second, we establish the relationship between the Rényi index and the volume-based dimension. Third, we analyze the meaning of the dimensions sequence, which characterizes the level of departure from the comparison benchmark based on the randomized time series. Finally, we use real stock market data from three countries for empirical analysis. In some cases, our proposed analysis method can more accurately capture the structural differences of networks than the power law index commonly used in previous studies.

## 1. Introduction

Many complex systems in the real world can be described using complex networks. In the last two decades, many concepts and algorithms have been proposed [1,2,3,4,5]. Since A. L. Barabási and R. Albert proposed a scale-free network model [6], the power law has become an important tool for characterizing network structures [7]. There are some dominant nodes in which the node degree is significantly larger than that of most nodes, and some researchers have discovered that there are other types of scaling laws in some networks that therefore define the dimensions of the network [8]. After the pioneering work of Song et al., other types of network dimensions were introduced [9,10,11,12,13,14,15,16,17]. For example, Daijun Wei et al. proposed the information dimension [9], and defined the Tsallis information dimension [10]. Rosenberg introduced the concept of maximal entropy minimal coverings to compute the information dimension [11]. O. Shanker defined the volume of a node in a network and introduced a new dimension based on the scaling law between the average of the volume and the distance [12,13]. The average density was defined by Guo Long et al., along with defining the dimension by the scale law of average density and distance [14].

The dimensions of weighted networks and the multifractal of networks have also been discussed by researchers [15,16,17]. In particular, researchers analyzed the multifractal of the network using the sandbox algorithm and found that there is multifractal in scale-free networks, but not in random networks [18]. A recent study shows that the heterogeneity of degree distribution is of crucial importance to the fractal properties of networks [19]. In addition to using the scaling law and information entropy to define network dimensions, some researchers also use the ergodic theory of dynamical systems to define the correlation dimension of a network [20].

In this article, we will apply the dimension proposed by O. Shanker to analyze a financial correlation-based network. We follow the terminology used by some researchers [21,22], and call the network constructed from the correlation matrix a correlation-based network.

At present, there are three usual methods for constructing correlation-based networks. One constructs the network using the minimum spanning tree (MST) algorithm [23]. The second method constructs the planar maximally filtered graph (PMFG), which can include some structures that are not in the MST, such as 4-clique and community [24]. The third approach constructs a 0–1 matrix based on a threshold and a correlation matrix, where the threshold is used to extract the structure in the correlation matrix [25,26]. For example, an element in a correlation matrix that is greater than a threshold is converted to 1, otherwise the element is converted to 0, so that a 0–1 matrix is generated as an adjacency matrix.

Since the work of Mantegna [23], many studies have explored applications of these methods, such as in stock markets [27], industrial indices [28], international indices, or foreign exchange markets [29,30]. Studies show that the degree distribution of some correlation networks satisfies the scale-free law [31,32,33,34,35,36]. Some researchers have found that the topological structure of the MST in the market changes over time, which leads to a change in the power law exponent of the degree distribution [34]. Further research has used the Rényi index to characterize the topological structural changes [36]. The Rényi Index is a standardized Rényi entropy used to characterize randomness and evenness; it has been correlated with the Lorenz curve [37,38]. Research shows that the Rényi index can effectively characterize the topological structure of MST, and this paper will analyze its relationship with the dimensions of a correlation-based network.

Many studies have applied entropy to the study of economic or financial issues [39,40]. For example, S.D. Bekiros constructed a discrete wavelet transformation based on entropy and used it to study currency returns [41]. R. Gencay and N. Gradojevic applied the maximum entropy principle to study the crash of 19 October 1987, and found the crash predictable [42]. Entropy has also been applied to the study of issues such as option pricing and asset pricing [43,44]. It can also be used to study financial hazards or predict systemic financial risks [45,46,47]. E. Maasoumi and J. Racine applied entropy metrics to study the predictability of returns in the stock market and found that the measure can detect nonlinear dependencies [48]. In particular, entropy has recently been applied to research networks and used to construct economic indicators of market fragility and systemic risk [49]. J. Yang and W. Qiu constructed a decision—making model based on entropy, and some problems that cannot be solved based on the mean—variance criterion can be dealt with [50].

In this article, we will sturdy the relationship between the dimensions of a network and the Rényi index. Our study is different from the application of entropy in finance used by most of the previous studies. This article discusses a correlation-based network, which leads us to focus on the correlation structure rather than a single financial temporal series. Recently, some researchers have applied the work of Song et al. to analyze the fractal of financial MST [51]. Based on this analytical framework, the MST of financial markets is non-fractal [51]. Here, we use the dimension proposed by O. Shanker to analyze the dimension of correlation-based networks, which is essentially a scale law defined on the basis of volume, with volume defined by the shortest distance. Then, we can directly calculate the shortest distance matrix and test whether there is a scaling law between volume and distance [12,13].

There are some differences between our calculation method and previous results [51]. First, we calculate the scaling law between volume and distance, whereas previous studies focused on the scaling law between the number of boxes and the distance. Secondly, we study the relationship between dimension and entropy, both commonly used indicators of complexity. Thirdly, to establish the relationship between these two indicators, we first generalize the definition of Shanker’s dimension and then use the data from three stock markets for empirical analysis.

The main finding of this paper is that the dimensions can be well defined on a correlation-based network and capture the details of changes in the network structure, and can therefore be used to study the dynamics of correlation-based networks.

## 2. Materials and Methods

### 2.1. Materials

We use daily closing price data from stock markets in China, the United Kingdom and the United States for empirical analysis. The Chinese data used in this paper are from the Wande database, while the data for the USA and UK markets are from Yahoo Finance.

Constituent stocks with missing data on the Chinese market for the Shanghai-Shenzhen 300 index (CSI300 index) were removed and a total of 162 stocks were selected from 4 January 2005 to 23 December 2015. Similarly, we exclude stocks with missing data in the Standard & Poor’s (S&P) 100 index between 3 January 2005 and 29 December 2014. In total, 93 stocks were selected. For comparative analysis, the daily closing price series of 80 constituent stocks of the Financial Times Stock Exchange (FTSE) 100 index were also used, excluding stocks with missing data, from 3 January 2005 to 29 December 2017.

### 2.2. MST and PMFG

We assume that there are *n* stocks V={1,…,n}, and that each stock *i* corresponds to a price time series $\left\{P_{i}\left(t\right)\right\}$. In the calculations of this paper, the stock price series needs to be preprocessed into a yield series: $\left\{R_{i}\left(t\right)\right\}$, where

(1) $$R_{i}\left(t\right)=\log\left(P_{i}\left(t+1\right)\right)-\log\left(P_{i}\left(t\right)\right).$$

The Pearson correlation coefficient between stocks *i* and *j* is calculated using

(2) $$\rho \left(i,j\right)=\frac{<R_{i}R_{j}>-<R_{i}><R_{j}>}{\sqrt{\left(<R_{i}^{2}-<R_{i}{>}^{2}><R_{j}^{2}-<R_{j}{>}^{2}>\right)}}.$$

The distance between stock *i* and *j* is defined as [23]:

(3) $$d\left(i,j\right)={\left(2-2\times \rho \left(i,j\right)\right)}^{\frac{1}{2}}.$$

In this paper, MST and PMFG are, respectively, calculated based on the distance matrix D=[d(i,j)] and the correlation coefficient matrix ρ=[ρ(i,j)]. We construct the MST using the classical Prim algorithm [52]. The minimum spanning tree is a planar graph with n−1 edges and no cycles, and with the minimum possible total edge weight. More details can be found in the literature [52].

Below, we briefly describe the construction of PMFG. The PMFG is also a planar graph that includes 3(n−2) edges, so that it contains 3-clique and 4-clique [24].

1. Pearson’s correlation coefficient between any two stocks *i* and *j* is calculated and denoted as ρ(i,j) (Equation (2)).
2. We extract elements of the upper triangular matrix of correlation coefficient matrix ρ=[ρ(i,j)] and arrange them in ascending order, denoted by $\rho_{sort}=\left\{\rho_{m}\left(i,j\right)\right\}$.
3. In order of $\rho_{sort}$, we add a link between the pairs of nodes of an element in $\rho_{sort}$ when the resulting graph is a planar graph.
4. The above step is repeated until a planar graph with 3(n−2) edges is generated.

In the following, each stock *i* corresponds to a node, so that the corresponding node is also labeled *i*.

### 2.3. Rényi Index

In general, for a network W(V,T), where matrix $T=[T_{ij}]$ is the adjacency matrix, the set V={1,2⋯n} is the node set. The degree of node *i* is $d_{i}=\sum_{j}T_{ij}$ and the average degree is $\bar{d}=\frac{1}{n}\sum_{i}d_{i}$. Further, the shortest distance matrix D=[D(i,j)] of the network *W* can be calculated, where D(i,j) is the length of the shortest path between nodes *i* and *j*.

In this paper, we will calculate the shortest distance matrix and construct different threshold networks. First, we need to denote the Heaviside step function as:

(4) H(t)=0,t≥0,

(5) H(t)=1,t<0.

Then, for a positive integer *r*, we can construct a threshold network $W_{r}\left(V,T_{r}\left(i,j\right)\right)$, where the element $T_{r}\left(i,j\right)=H\left(D\left(i,j\right)-r\right)$. That is, the elements in the shortest distance matrix that are less than *r* are converted to 1, otherwise to 0. Here, it is assumed that $T_{r}\left(i,i\right)=0$. The degree of node *i* and the average of the threshold network are, respectively, denoted $d_{i}\left(r\right)=\sum_{j}T_{r}\left(i,j\right)$ and $d_{m}\left(r\right)=\frac{1}{n}\sum_{i}d_{i}\left(r\right)$.

The Rényi index is a standardized Rényi entropy, which can be used to characterize randomness and evenness [37]. Consider a human population consisting of *n* members, each of which owns wealth $w_{i}$ and thus has a wealth vector $w=(w_{1},w_{2},\ldots w_{n})$. Then, the Rényi index of the wealth vector *w* is defined as [37]

(6) $$R\left(q\right)=1-{\left\{\sum_{i=1}^{n}{\left(\frac{w_{i}}{\bar{w}}\right)}^{q}\times \left(\frac{1}{n}\right)\right\}}^{\frac{1}{1-q}},q\neq 1,$$

(7) $$R\left(1\right)=1-\exp\{-\sum_{i=1}^{n}\left(\frac{w_{i}}{\bar{w}}\times \ln\left(\frac{w_{i}}{\bar{w}}\right)\times \frac{1}{n}\right)\},q=1,$$

where $\bar{w}=\frac{1}{n}\sum_{i}w_{i}$ is the average wealth and *q* is a parameter.

Further research has found that the Rényi index can be effectively applied to characterize the topological structure of financial MST [36]. In general, we can define the Rényi index of network *W* as

(8) $$R\left(q\right)=1-{\left\{\sum_{i=1}^{n}{\left(\frac{d_{i}}{\bar{d}}\right)}^{q}\times \left(\frac{1}{n}\right)\right\}}^{\frac{1}{1-q}},q\neq 1,$$

(9) $$R\left(1\right)=1-\exp\{-\sum_{i=1}^{n}\left(\frac{d_{i}}{\bar{d}}\times \ln\left(\frac{d_{i}}{\bar{d}}\right)\times \frac{1}{n}\right)\},q=1,$$

where the degree of node *i* is analogous to wealth.

Next, we study the relationship between the heterogeneity and dimension of correlation-based networks. Naturally, the Rényi index R(q,r) (q≠1) of the threshold network $W_{r}$ can also be calculated as

(10) $$R\left(q,r\right)=1-{\left\{\frac{\sum_{i}d_{i}{\left(r\right)}^{q}}{d_{m}{\left(r\right)}^{q}}\times \frac{1}{n}\right\}}^{\frac{1}{1-q}}.$$

### 2.4. Dimension

Since MST and PMFG are always connected networks, we can directly calculate the shortest distance between any two nodes. Based on the dimension proposed by O. Shanker [12], the calculation steps are as follows:

1. We calculate MST or PMFG based on distance matrix or correlation coefficient matrix. Here, the correlation-based network is denoted as W(V,T), where V={1,2,...,n} is a node set and T=[T(i,j)] is an adjacency matrix.
2. The shortest distance matrix $D_{s}=\left[D_{s}\left(i,j\right)\right]$ is calculated by the adjacency matrix *T*.
3. We set the threshold set $L=\{l_{s},s=1\ldots k\}$ and then compute threshold network $W_{l_{s}}\left(V,T_{l_{s}}\right)$ for $l_{s}$, where the elements of $T_{l_{s}}\left(i,j\right)=H\left(D_{s}\left(i,j\right)-l_{s}\right)$.
4. The number of non-zero elements in the *i*-th row of matrix $T_{l_{s}}$ is the volume of node *i* with distance $l_{s}$. That is, the volume $V_{i}\left(r\right)$ of node *i* is its degree in the threshold network $W_{l_{s}}$. Further, the volume $V(l_{s})$ is calculated using
  (11) $$V\left(l_{i}\right)=\sum_{k}V_{k}\left(l_{i}\right)/n,$$
  that is, the average is calculated.
5. If the scaling relationship is as
  (12) $$V\left(r\right)∼Cr^{D_{V}},$$
  the volume dimension $D_{V}$ is defined, where *C* is a constant and *r* is the distance.

In the calculation, we need to select the appropriate set *L* and then estimate $V_{d}$ in the double logarithmic coordinate system by

(13) $$log\left(V\left(l_{s}\right)\right)=C_{0}+d_{V}\times \log\left(l_{s}\right),$$

where $C_{0}$ is a constant.

### 2.5. Generalized Volume-Based Dimensions

In this section, we will define the volume dimension in a generalized way based on the concept of volume. We note that the volume V(r) is the average of the volume of all nodes *i* ($\left\{V_{i}\left(r\right),i=1\cdots n\right\}$, Equation (11). In general, we define V(r,q) as

(14) $$V\left(r,q\right)={\left(\sum_{i}V_{i}{\left(r\right)}^{q}/n\right)}^{\frac{1}{q}},q\geq 1,$$

where q≥1. When *q* is a positive integer, the expression $\sum_{i}V_{i}{\left(r\right)}^{q}/n$ is the *q*-th sample moment of the volume $\left\{V_{i}\left(r\right)\right\}$. For any real number q≥1, the latter calculation shows that there is still a scale relationship between V(r,q) and distance *r*. As in the definition of dimension proposed by O. Shanker [12], we define the generalized dimensions as follows: if there is a scaling relationship between V(r,q) and distance *r* as

(15) $$V\left(r,q\right)∼Con_{q}r^{D_{V,q}},q\geq 1,$$

where *r* is the threshold, $Con_{q}$ is a constant, then the index $D_{V,q}$ is a generalized dimension. As a special case, when q=1, $D_{V,1}=D_{V}$. For a set of suitable thresholds $\left\{l_{s}\right\}$, $D_{V,q}$ can be fit using a least square method, as follows (*C* is a constant):

(16) $$log\left(V\left(l_{s},q\right)\right)=C+D_{V,q}\times \log\left(l_{s}\right).$$

The generalized dimension can be used to study higher-order statistics of volume sequence $\left\{V_{i}\left(r\right),i=\ldots n\right\}$ and is naturally embedded in the definition of the Rényi index. Since the volume $V_{i}\left(r\right)$ is the degree of node *i* ($d_{i}\left(r\right)$) in the threshold network $W_{r}$,

(17) $$R\left(q,r\right)=1-{\left(\frac{V{\left(r,q\right)}^{q}}{V{\left(r,1\right)}^{q}}\right)}^{\frac{1}{1-q}},q>1,$$

can be obtained from Equations (10) and (14).

To further simplify Equation (17),

(18) $$R\left(q,r\right)=1-C\times r^{\frac{q(D_{V,q}-D_{V,1})}{1-q}},C={\left(\frac{Con_{q}}{Con_{1}}\right)}^{\frac{q}{1-q}},q>1,$$

is introduced, where *C* is a constant. If $D_{V,q}=D_{V,1}$, then R(q,r)=0, which means that the degree of nodes in the network is homogeneous. Based on Equation (18), the difference between the dimensions can also be expressed by the Rényi index. In particular, the original volume-based dimension ($D_{V,q}=D_{V,1}$) is the basic dimension, with which other dimensions are compared to characterize the Rényi index as follows:

(19) $$D_{V,q}-D_{V,1}=\frac{1-q}{q}\times \frac{log(1-R(q,r))-log(C)}{log(r)},C={\left(\frac{Con_{q}}{Con_{1}}\right)}^{\frac{q}{1-q}},q>1.$$

When r=2, Equation (18) allows the Rényi index of the original network W(V,T) to be expressed in terms of dimensions, as

(20) $$R\left(q,2\right)=1-C\times 2^{\frac{q(D_{V,q}-D_{V,1})}{1-q}},C={\left(\frac{Con_{q}}{Con_{1}}\right)}^{\frac{q}{1-q}},q>1.$$

However, *r* can take any value in the set of thresholds ($\left\{l_{s}\right\}$), so the dimension also expresses the Rényi index of the threshold network $W_{r}$. Since the degree of node *i* in the threshold network expresses the more neighbor information of the node, the dimension contains more information of the network structure.

## 3. Results

### 3.1. Generalized Volume-Based Dimensions

**Figure 1:**
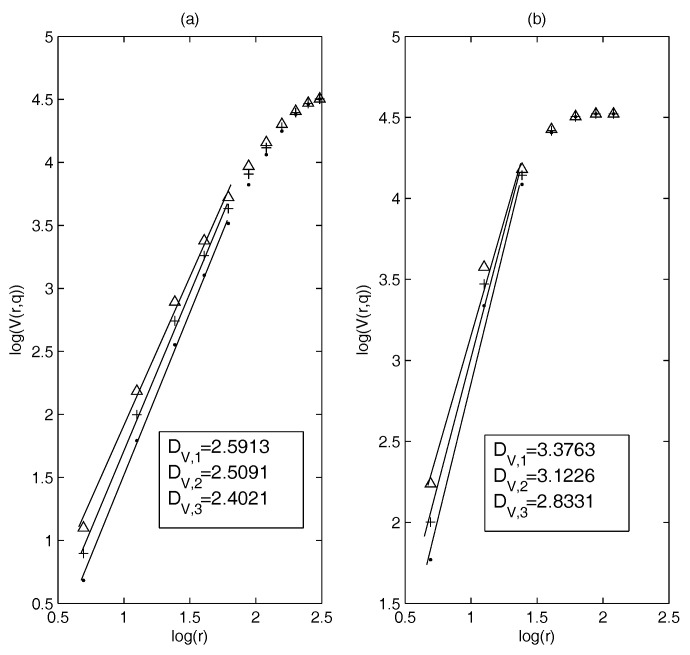
The dimension estimation of: (**a**) minimum spanning tree (MST); and (**b**) planar maximally filtered graph (PMFG) (point: $D_{V,1}$, +: $D_{V,2}$, triangle: $D_{V,3}$).

First, we calculate MST and PMFG based on the constituent stocks of the S&P 100 index (3 January 2005–29 December 2014) and estimate the dimensions. Figure 1a,b show the results of the dimension estimation: Figure 1a corresponds to MST and Figure 1b to the PMFG. It can be seen that in a suitable threshold interval the relationship between log(V(r,q)) and log(*r*) is nearly linear. This means that in this example, the dimensions can be defined well on MST and PMFG. We choose L={2,3,4,5,6} and L={2,3,4} when estimating the dimensions of MST and PMFG, respectively. Calculations show that the generalized volume-based dimensions can be well defined on the correlation-based networks.

In our study, we find that the threshold sets L={2,3,4,5,6} and L={2,3,4} are suitable for estimating the dimensions of MST and PMFG, respectively. Therefore, the later threshold set in the calculation is consistent with this example.

Next, to establish a benchmark for comparative analysis, we randomize the yield series and calculate the correlation-based network, then estimate the dimensions and compare them with the dimensions based on the real data. We still choose the data used in Figure 1.

Now, the series of yield for each stock is randomly reordered. Note that this step does not change the distribution of yield. Then the correlation coefficient matrix and distance matrix between the yield series are calculated. Finally, we calculate MST and PMFG and estimate their dimensions.

**Figure 2:**
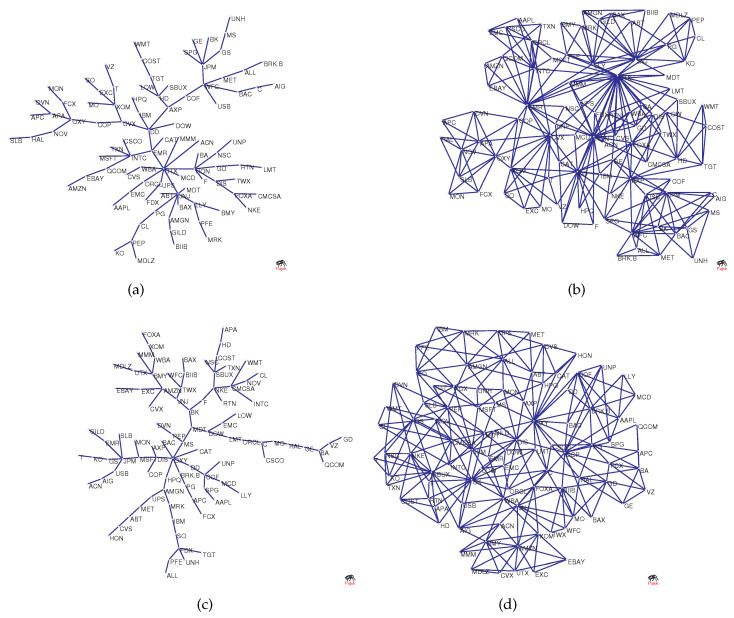
(**a**,**b**) The original networks; and (**c**,**d**) the benchmark networks used for comparison.

To visualize the structural changes in the network, Figure 2a–d shows the original network versus the network as a benchmark. The figure shows MST and PMFG based on real data, where the maximum degree (UTX) in Figure 2a is 9, and the maximum degree (UTX) in Figure 2b is 30. However, the degree of node UTX in Figure 2c is 2 and in Figure 2d it is 5. This is because randomized series eliminate the original correlation structure, causing hub nodes to lose their core positions.

**Figure 3:**
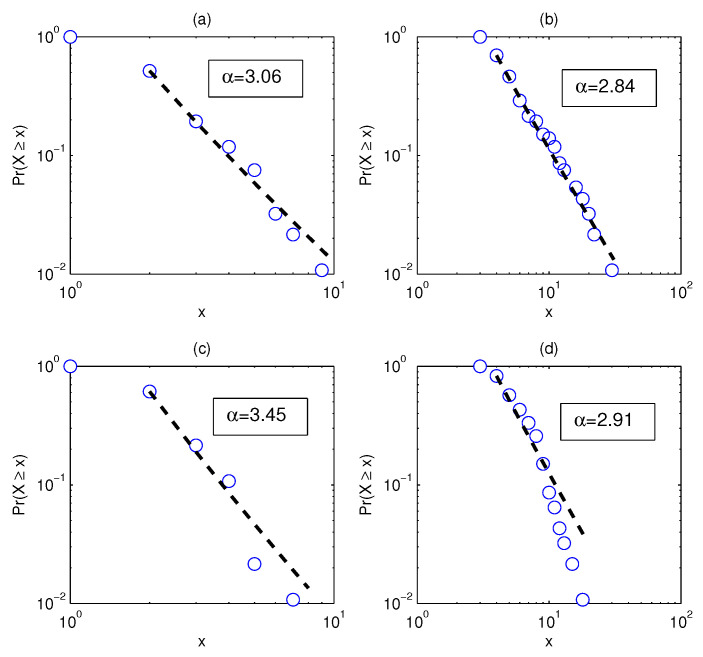
The degree distribution of the network in Figure 2, in which the labels of the four subgraphs correspond to the labels in Figure 2 one by one, for example, Figure 3a corresponds to Figure 2a and so on. (**a**) α=3.06; (**b**) α=2.84; (**c**) α=3.45; (**d**) α=2.91.

The structural changes directly lead to the changes in the degree distribution. Figure 3a–d shows the degree distributions of the four networks in Figure 2. It is assumed here that the degree distribution satisfies the power law $p\left(x\right)∼x^{-\alpha }$. We compare Figure 3a,c and find that the power law exponent of the latter is larger, which implies that the degree distribution is less heterogeneous.

**Figure 4:**
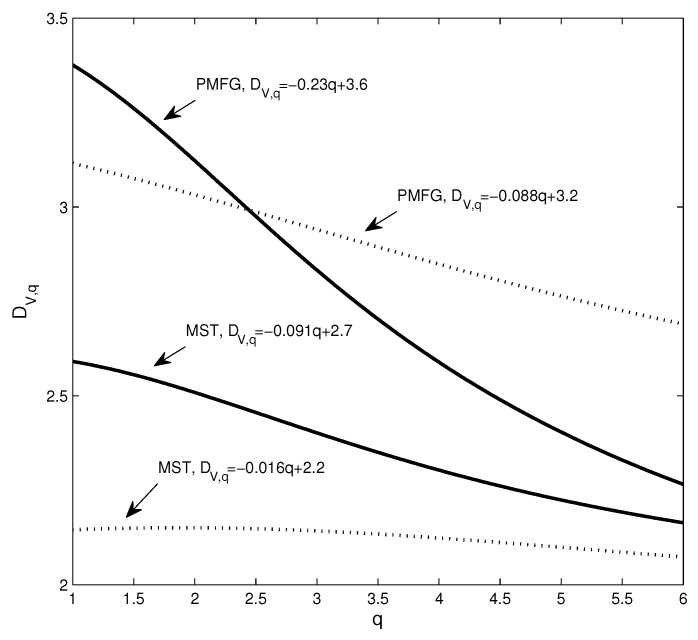
The dimension sequences of the different networks, where the solid line corresponds to the real data and the dotted line corresponds to the randomized data.

Next, we use the dimension and Rényi index to analyze the changes in the structure of the network. Figure 4 shows the estimation of the dimensions when taking different *q* values, where the solid line corresponds to the network based on real data and the dotted line corresponds to the network based on the randomized series. Intuitively, it can be found that the change of the dotted line is smoother. In addition, we calculate the Rényi index index for each network. Corresponding to the four subgraphs in Figure 3a–d, the Rényi indices are 0.3476 (Figure 2a), 0.3715 (Figure 2b), 0.2352 (Figure 2c), and 0.1885 (Figure 2d).

To show the changes of the four dimension sequences in Figure 4 more clearly, the four data sets are approximated by straight lines. It can be seen that the absolute value of the coefficient of *q* corresponding to the solid line is significantly larger than the coefficient of the benchmark-based *q*. For example, the absolute value of the coefficient of *q* corresponding to the PMFG of the real data is 0.23, which is more than 2.6 times 0.088. In particular, the differences in the structure of some networks cannot be accurately captured by the power law exponent. The power law exponent of the networks shown in Figure 2b,d is 2.84 and 2.91, respectively. The difference between the two power law exponents is small, yet Figure 4 shows that there is a significant difference between the two networks. We find that the Rényi index and the dimensions can clearly and quantitatively show the structural changes.

Below, we analyze the results based on a factor model. In modern finance theory, multi-factor models are often used to model stock returns [53]. For example, the three-factor model is one of the most commonly used multifactor models [54]. More recently, some researchers have also used factor models to study networks, such as constructing factor models to analyze network structures [55], or applying a three-factor model to studying network-based portfolios [56]. In the factor model, in general, a normalized series of returns can be expressed as a linear combination of *m* factors, as shown in [57]

(21) $$R_{i}\left(t\right)=\sum_{k}^{m}\beta_{ik}f_{k}\left(t\right)+\varepsilon_{i}\left(t\right).$$

In Equation (21), the $\beta_{ik}$ are the linear exposure of the variable $R_{i}\left(t\right)$ to the factor $f_{k}\left(t\right)$ (k=1,⋯,m) at time *t* and the $\varepsilon_{i}\left(t\right)$ is the idiosyncratic part of $R_{i}\left(t\right)$ ($E(\varepsilon_{i}\left(t\right))=0$).

Furthermore, Equation (21) can be re-expressed as a matrix form, as shown in

(22) R=βF+ε,

where $R={\left[R_{1}\left(t\right),\cdots ,R_{n}\left(t\right)\right]}^{t}$, $\beta ={\left[\beta_{i,k}\right]}_{n\times m}$, $F={\left[f_{1}\left(t\right),\cdots ,f_{m}\left(t\right)\right]}^{t}$, and $\varepsilon ={\left[\varepsilon_{1}\left(t\right),\cdots ,\varepsilon_{n}\left(t\right)\right]}^{t}$ [57]. Here, *t* represents the transpose of the matrix. Thus, we can express the covariance matrix of a set of yield series as shown in

(23) $$C=\beta C_{F}\beta^{t}+C_{\varepsilon },$$

where $C_{F}$ is the covariance matrix of the factors $\left\{f_{k}\left(t\right),k=1,\cdots ,m\right\}$ and $C_{\varepsilon }$ is the covariance matrix of the residuals $\varepsilon_{i}\left(t\right)$ [57].

The covariance matrix of the normalized series is the correlation coefficient matrix of the yield series. Equation (23) means that the matrix of correlation coefficients can be linearly represented by some factors. In our study, when the yield series are randomized, the yield series are no longer factor driven. As a result, the hub node is converted to a non-hub node, as shown in Figure 2. Structural changes can be captured by the Rényi index and the dimension sequence, as shown in Figure 4. In general, when the structural changes of the network, such as from the star to the chain, the Rényi index also changes, based on Equation (19), the dimension sequence changes.

In summary, we can characterize the differences between the networks and their benchmarks by analyzing the curvature of dimension sequences. Since a network for the comparison benchmark is generated based on a randomized time series, it can be considered as having no notable structure. Thus, the more dramatic changes in the dimension sequence, the higher the deviation from the benchmark. Therefore, the sequence of dimensions characterizes the complexity of the network. In addition, we construct the relationship between the Rényi index and the dimensions, as shown in Equations (18) and (19). This means that the dimension sequence also contains information about the structure of the threshold network, whereas the original network can be considered as a special threshold network (r=2). In the next section, we will study the relationship between the dimension and the Rényi index of the threshold network.

### 3.2. Relationship between the Dimension and the Rényi Index of the Threshold Network

**Figure 5:**
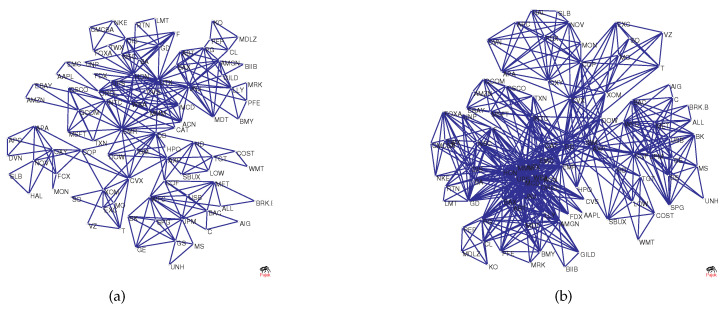
The figure shows two threshold networks based on Figure 2a: (**a**) $W_{3}$; and (**b**) $W_{4}$.

In the previous section, Equation (18) implied that the dimension not only contains the information of the structure of the original network but also the information in the threshold network $W_{r}$. We select the network in Figure 2a as an example to generate the threshold networks $W_{3}$ and $W_{4}$, as shown, respectively, in Figure 5a,b. Intuitively, we can see that there is a significant difference between $W_{3}$ and $W_{4}$, and network $W_{4}$ includes even more edges. Network $W_{3}$ includes second-order information of a node, that is, other nodes at a distance of 2 from the node are regarded as neighbors, and network $W_{3}$ includes third-order information.

We will next show that the dimension contains information about the structure of networks $W_{3}$ and $W_{4}$. Based on Equation (18), R(2,3) and R(2,4) are estimated using the dimensions $D_{V,1}$ and $D_{V,2}$ estimated in Figure 1a, respectively. In addition, The Rényi index of the threshold network can be calculated directly using Equation (10), denoted as R(2,r). The comparison results are shown in Table 1. In Table 1, the Rényi index calculated based on dimensions is denoted as $R^{\prime }\left(2,r\right)$. Calculations show that the Rényi index value of $W_{4}$ is less than the Rényi index value of $W_{3}$, which means its degree distribution is more homogeneous. It can also be seen that the difference between $R^{\prime }\left(2,r\right)$ and R(2,r) is small.

In summary, our analysis shows that the dimension sequence includes information on the structure of the threshold network.

### 3.3. Empirical Analysis Based on Different Countries

In this section, we use data from three stock markets for analysis. We choose a special case to show the relationship between the dimension and the Rényi index. We set q=1 and q=2 to calculate the dimension series, and q=2 to calculate the Rényi index series. For convenience, we define

(24) $$Diff_{dim}\left(2,1\right)=D_{V,2}-D_{V,1}$$

in the following subsections.

During the period considered, the number of trading days in the UK and China markets were approximately 253 and 242, respectively. In this section, we only set the length of the calculation window to 126 days, which is about half the number of trading days in the USA market. In the following, the dimension series $D_{V,1}$, $D_{V,2}$ and Rényi series are calculated simultaneously, where the calculation window is 126 days and the sliding window is 1 day. Then, the difference between $D_{V,1}$ and $D_{V,2}$ is calculated $Diff_{dim}\left(2,1\right)$ for each time period.

**Figure 6:**
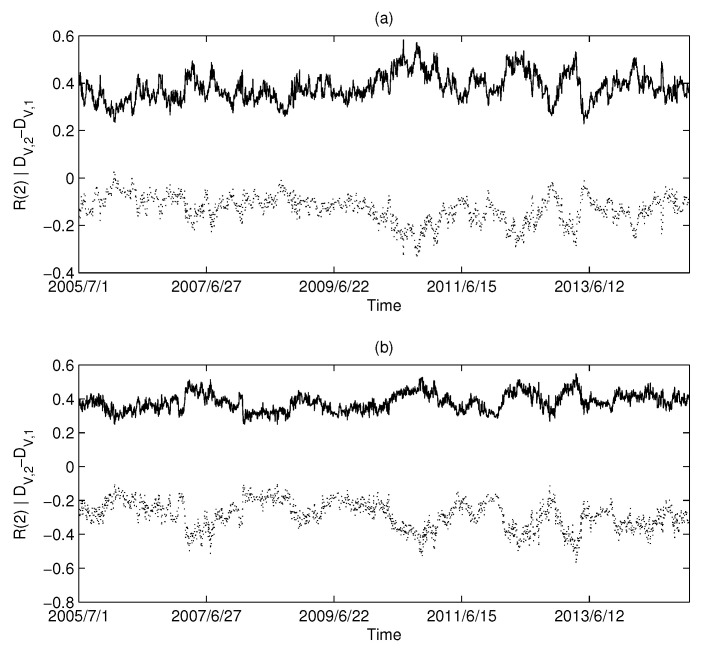
The dimension and Rényi index series of the correlation-based networks in the USA market: (**a**) MST; and (**b**) PMFG. The solid line corresponds to the Rényi index, and the dotted line corresponds to $Diff_{dim}\left(2,1\right)$.

The calculation results of the USA market are shown in Figure 6a,b: Figure 6a corresponds to MST and Figure 6b corresponds to PMFG. Intuitively, we can find that there is a highly negative correlation between the $Diff_{dim}\left(2,1\right)$ and Rényi series in the USA market.

**Figure 7:**
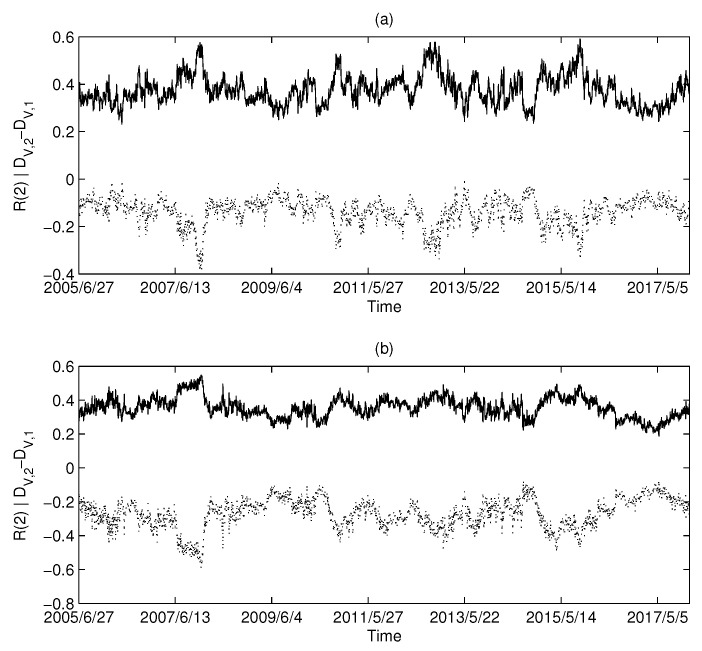
The dimension and Rényi index series of the correlation-based networks in the British market: (**a**) MST; and (**b**) PMFG. The solid line corresponds to the Rényi index, and the dotted line corresponds to $Diff_{dim}\left(2,1\right)$.

**Figure 8:**
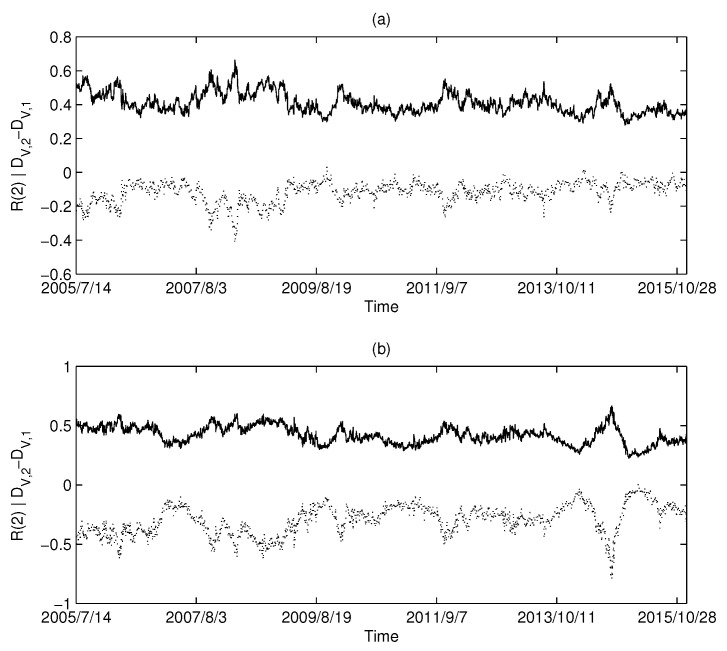
The dimensionality and Rényi index series of the correlation-based networks in the Chinese market: (**a**) MST; and (**b**) PMFG. The solid line corresponds to the Rényi index, and the dotted line corresponds to $Diff_{dim}\left(2,1\right)$.

Similarly, the calculated results using UK market data and China market data are shown in Figure 7 and Figure 8, respectively. Both the UK and China markets showed similar results to the USA market. Intuitively, we find that there is a synchronization between the $Diff_{dim}\left(2,1\right)$ series and the Rényi index series. We calculated the Pearson correlation between each pair of $Diff_{dim}\left(2,1\right)$ series and the Rényi series and are listed in Table 2. The high level of correlation shown in the calculations is consistent with the results predicted by Equations (18) and (19).

### 3.4. Robust Analysis of Calculation Window

**Figure 9:**
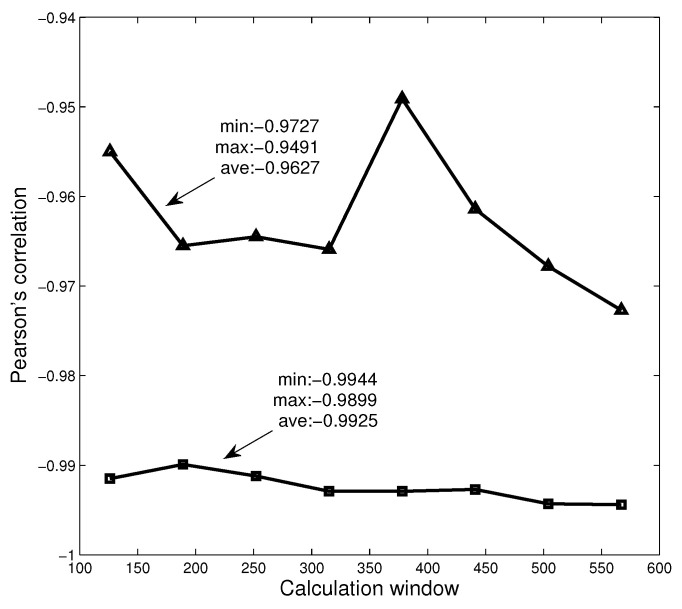
The figure shows the relationship between the Pearson correlation coefficient and the calculation window.

In the previous section, we have analyzed the relationship between the Rényi index series and the dimension series of networks in three different markets. In the analysis, we set the calculation time window to be 126 days. To further study the robustness of the calculation window, we set different time windows and study the relationship between Rényi index and dimension. Here, we choose the data of American market and set eight windows (k×63 days, k=2,⋯,9), respectively, to calculate Rényi index series and $Diff_{dim}\left(2,1\right)$ series. The calculation results are shown in Figure 9, where the triangles and squares correspond to MST and PMFG, respectively. It can be found that the Pearson correlation coefficients corresponding to all time windows are less than −0.94, and the average values corresponding to MST and PMFG are all less than −0.96. The calculation results show that the time window does not change the conclusion that there is a high correlation between Rényi index and $Diff_{dim}\left(2,1\right)$ series.

### 3.5. The Dynamics of the Rényi Index

**Figure 10:**
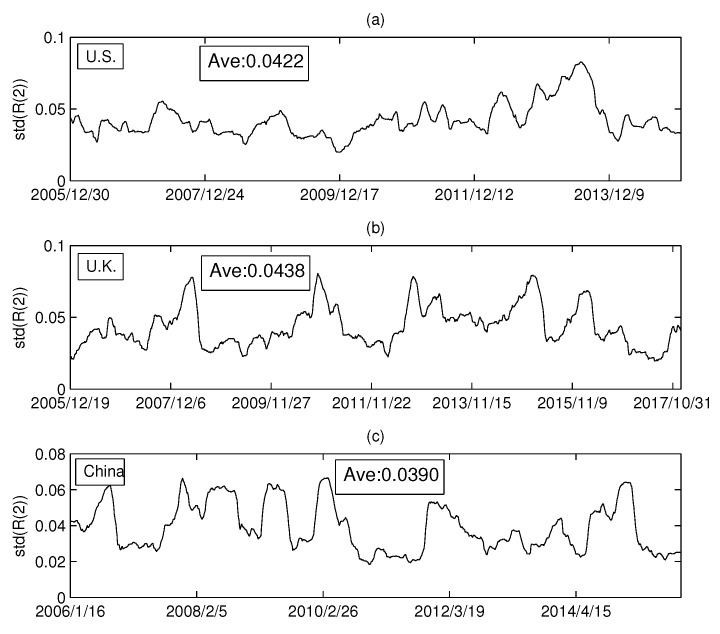
The series of standard deviations (MST): (**a**) the USA market; (**b**) the UK market; and (**c**) the China market.

**Figure 11:**
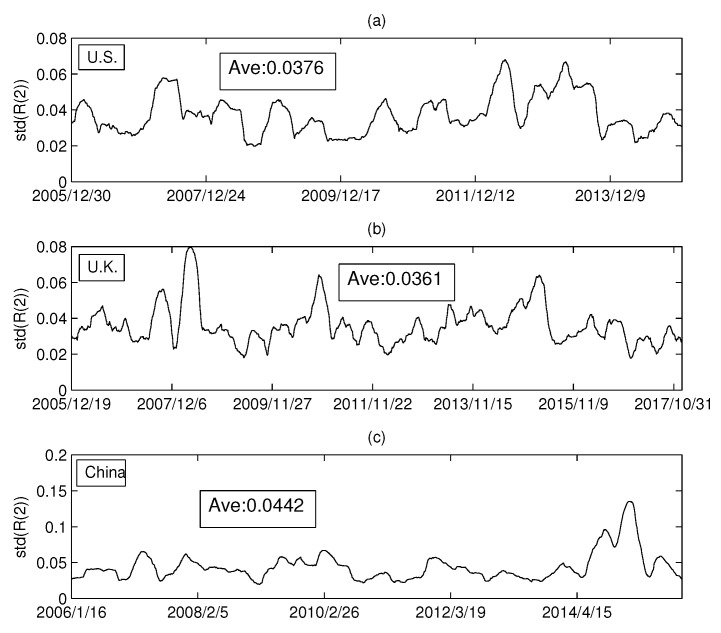
The series of standard deviations (PMFG): (**a**) the USA market; (**b**) the UK market; and (**c**) the China market.

In the previous section, we empirically analyzed the relationship between the Rényi index and the volume dimension. Intuitively, the Rényi index was found to vary drastically over time. To analyze the dynamics of the Rényi index in more detail, this section will calculate the standard deviation of the Rényi series for the three markets. We set the calculation time window at 126 days, and the sliding window at one day. In this way, a standard deviation series of Rényi series is calculated. Figure 10a–c shows the standard deviations of the MST Rényi index for the U.S. market, the U.K. market, and the China market, respectively. Similarly, Figure 11a–c shows the standard deviation series of the Rényi index for PMFG.

We calculated the average of each series in Figure 10 and found that the difference between the three markets was small. However, Figure 11 shows that the Rényi index of PMFG in China fluctuates significantly more than that of the other two markets. The dramatic change in the index over a period of time can lead to an increase in the standard deviation. Comparing the Chinese market with the other two markets, we find that the Rényi index in the Chinese market changed drastically from 2014 to 2015, as shown in Figure 11c. A more detailed analysis shows that the mean of the data up to 30 June 2014 is 0.0392. The difference between 0.0392 and the average of the other two markets (0.0376 and 0.0361) is not significant. Therefore, the difference between the Chinese market and the other two markets is mainly due to the data changes from July 2014 to June 2015. During this period, a huge bubble was generated in the Chinese market and was broken in June 2015. Taking the CSI 300 Index as an example, the index on 1 July 2014 was 2164.559 and reached the peak of 5353.751 on 8 June 2015. After that, the bubble broke down and the index dropped drastically to 4253.021 on 1 July 2005. In addition, the maximum (0.1351) in Figure 11c reached on 2 March 2015, and then rapidly decreased to 0.0299 (on 12 June 2015).

We have found in Section 2.1 that there is no hub node in the network based on the randomized sequence, resulting in a decrease of the Rényi index. Similarly, in previous studies, researchers found changes in the network structure, such as the central company changes as the marginal company, leading to power law exponent changes [34]. Our research also shows that the drastic change in the number of central firms that correspond to the core nodes leads to a change in the Rényi index, as shown in Figure 2 and Figure 3. The drastic change in the Rényi index also suggests that the relationship between companies changes significantly over time, leading to more unstable structures. Therefore, the results shown in Figure 11 show that the changes in PMFG in the Chinese market may be related to this structural change. Furthermore, since the time series can be explained by a multifactor model, as shown in Section 2.1, we speculate that the underlying causes of this change are due in part to changes in economic factors in the Chinese market. The market index has fluctuated dramatically during the period when the bubble was generated and broken. The economic factors that drive the price changes of stocks during this period may have changed, leading to differences between the Chinese market and the other two markets.

### 3.6. Example of Volume-Based Dimension Analysis

The calculations in the previous section show that the structure of the network varies drastically over time. In this section, we will examine a concrete example using the analytic framework of dimension-entropy.

**Figure 12:**
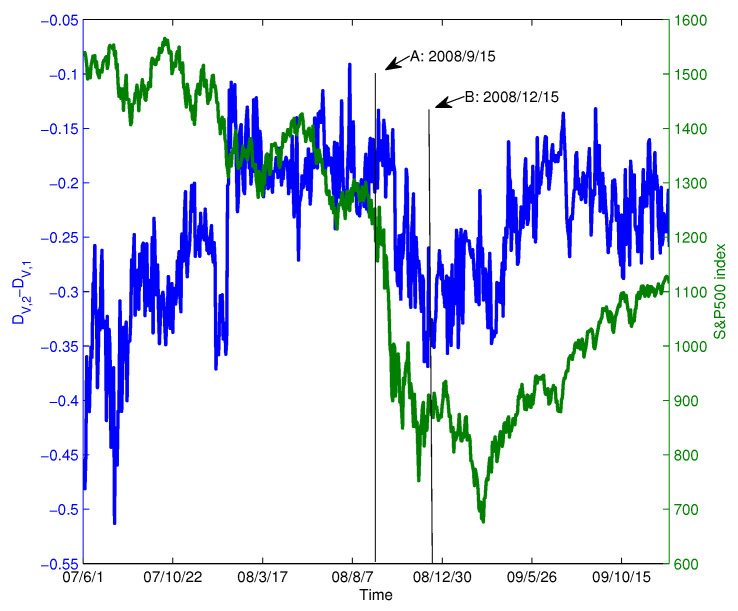
The $Diff_{dim}\left(2,1\right)$ series and the S&P 500 index, where Point A corresponds to the collapse of Lehman Brothers, while Point B corresponds to 15 December 2008.

**Figure 13:**
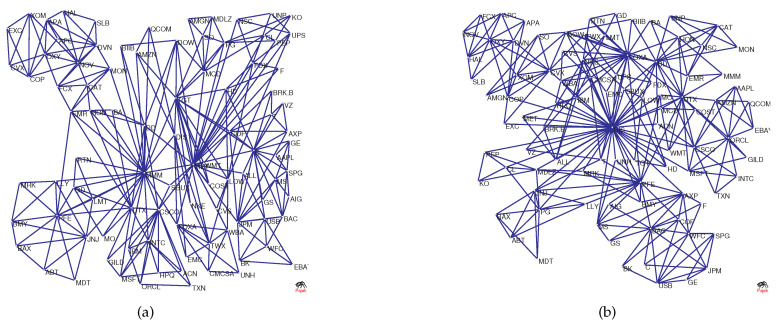
The PMFG corresponding to Points A and B: (**a**) the collapse of Lehman Brothers; and (**b**) 15 December 2008.

During the financial crisis of 2008, the collapse of Lehman Brothers was an important event. We chose the data of constituent stocks in the S&P100 index from 1 June 2007–31 December 2009 for analysis. We extract the data for this period from Figure 6b and show it in Figure 12 (blue line). For comparison, the S&P 500 index is also shown in Figure 12 (green line). In the figure, Point A corresponds to the collapse of Lehman Brothers on 15 September 2008. Overall, we find that as the index decreases drastically, $Diff_{dim}\left(2,1\right)$ also changes drastically and declines from 15 September–15 December 2008. At Point A, the value of $Diff_{dim}\left(2,1\right)$ is −0.1850; however, after that, the value of $Diff_{dim}\left(2,1\right)$ varies dramatically and reaches −0.3445 after three months. This implies that the structure of the PMFG varies significantly. To visualize this change, Figure 13a,b shows the PMFGs for Points A and B.

Comparing Figure 13a with Figure 13b, we find that there is a super hub node in Figure 13b, which has a degree of 43, whereas, in Figure 13a, the maximum degree is 25.

**Figure 14:**
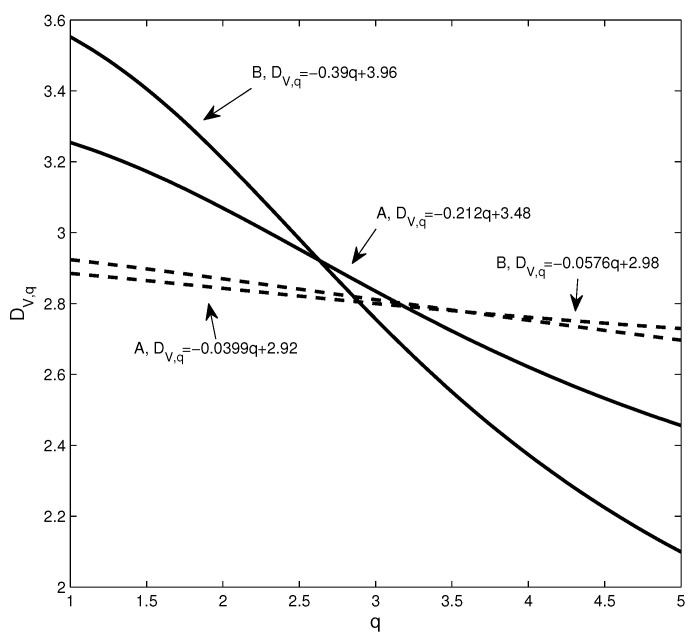
The dimension sequence corresponding to the two points in Figure 11; the solid and dotted lines correspond to the real data and the benchmark, respectively.

This structural change can be well captured using the dimension sequence, as shown in Figure 14. The changes in the dimension sequences corresponding to the two networks are significantly different. For comparison, we randomize the yield series for the time period and construct the PMFG according to the method of generating the benchmark shown in Section 2.1.

We find three differences between the dimension sequences at Points A and B. First, the corresponding dimension ($D_{V,1}$) of Point B is greater than Point A, which means that as the distance increases, the volume changes more rapidly. Second, the solid line corresponding to Point A changes more smoothly, which means that the PMFG at Point B deviates farther from the benchmark. Third, to show the difference between different dimension sequences, we use a line to fit the data to get the relationship between dimension and *q* as shown in the figure. It can be seen that the PMFGs at Points A and B are all significantly different from the benchmark, and the difference at Point B is greater.

In summary, we find that the market’s correlation structure changed drastically and deviated significantly from the benchmark. This also suggests that the complexity of the correlation structure in the market changes over time, especially during a financial crisis.

## 4. Discussion and Conclusions

### 4.1. Discussion

In our study, both the dimension and the Rényi index are defined on an undirected network, which are used to extract the structure in the Pearson correlation matrix. At present, some research focuses on the networks constructed by other methods, for instance constructing partial networks by using partial correlation coefficients or constructing causal networks [58,59,60]. One area of further possible study is to discuss the dimension and Rényi index on these directed networks.

In this article, we use the method of rolling time windows to construct the network. Recently, researchers have estimated the dynamic correlation between time series and constructed networks that can avoid rolling time windows; however, it is difficult to estimate and construct larger networks [61,62]. Therefore, further research should focus on networks based on dynamic correlation.

Here, the dynamics of dimension and Rényi index have been studied, but its mechanism needs further study. First, in Section 2.1, we use a multifactor model to explain the change in the correlation structure caused by the randomized time series, and this change is captured by the Rényi index and the dimension sequence. Second, in Section 2.3 and Section 2.5, calculations show that the dimension series and the Rényi index change over time in different markets, whereas the Rényi index of the PMFG in the Chinese market changes more drastically. On the one hand, the yield series can be directly expressed as a linear combination of factors. On the other hand, the change of network structure can be affected by the change of factors, as shown in Section 2.1. Therefore, it is necessary to further study the mechanism explanation of network structure changes based on economic factors. Further research may need to focus on the influence of the factors on the network structure, as well as on the dynamics of Rényi index and dimension.

### 4.2. Conclusions

In studying the relationship between the dimensions of the correlation network and the Rényi Index, using the data of three markets for empirical analysis, we find that volume-based dimension is well defined on a correlation-based network. Our studies have shown that there is also a scaling relationship between the higher moment of the volume and the distance. Based on this empirical fact, we constructed a general volume-based dimension. We also find that the volume-based dimensions are intrinsically linked to the network’s Rényi index.

Our analysis results show that the dimensions can reveal the topological structure of the network well and include the neighbor information of the nodes. Volume-based dimension sequences characterize the level of deviation from the benchmark based on randomized series, thus describing the complexity of correlation-based networks. In addition, our analytical framework may also be applied to complex systems, such as those in financial markets, where each element can be characterized by time series, and the relationships among the different elements can be constructed based on the correlation.

## Figures and Tables

**Table 1:** The Rényi index of the threshold network.

Figure	$D_{V,1}$	$D_{V,2}$	*C*	*r*	$R^{\prime }\left(2,r\right)$	R(2,r)
Figure 5a	2.5913	2.5091	0.5658	3	0.3221	0.3362
Figure 5b	2.5913	2.5091	0.5658	4	0.2893	0.2842

**Table 2:** Pearson’s correlation coefficient (PCC) between the $Diff_{dim}\left(2,1\right)$ series and the Rényi index series (q=2).

Figure	Figure 6a	Figure 6b	Figure 7a	Figure 7b	Figure 8a	Figure 8b
PCC	−0.9550	−0.9915	−0.9374	−0.9869	−0.9197	−0.9859
